# Grey-scale ultrasound findings of lower extremity entheses in healthy children

**DOI:** 10.1186/s12969-015-0012-1

**Published:** 2015-05-06

**Authors:** Clara Lin, Mohammad Diab, Diana Milojevic

**Affiliations:** Children’s Hospital Colorado Pediatric Rheumatology, 13123 East 16th Street, Box B311, Aurora, CO 80045 USA; University of California-San Francisco Pediatric Orthopedic Surgery, 400 Parnassus Ave, San Francisco, CA 94143 USA; Floating Hospital for Children @ Tufts Medical Center, 800 Washington Street #190, Boston, MA 02111 USA

**Keywords:** Ultrasound, Sonography, Enthesitis-related arthritis, Entheses, Spondyloarthropathy, Osteochondritis

## Abstract

**Background:**

To describe grey-scale sonographic findings in lower extremity entheses in healthy children.

**Methods:**

Healthy patients referred to Orthopedic Surgery or Adolescent Medicine outpatient clinics or their siblings ages 5-18 years were recruited. Grey-scale ultrasound was performed on 3 entheseal sites bilaterally, the proximal patellar ligament insertion (PPL), distal patellar ligament insertion (DPL), and Achilles tendon insertion (AT). Entheseal thickness and quality were recorded. Comparison of thickness between contralateral sites was evaluated to determine within subject site variability.

**Results:**

702 entheses were examined in 117 children. Age had a weak positive correlation with thickness with large variability. Weight had the strongest correlation to thickness. Contralateral sites are comparable in thickness; a difference of 28%, 26%, and 18% between bilateral PPL, DPL, and AT, respectively, falls within the 95^th^ percentile of the healthy pediatric population in this study. The patellar ligament contour evolved with age from a curved to linear contour.

**Conclusions:**

Weight is the best predictor of entheseal thickness in children although there is a large degree of variability. Contralateral entheses are comparable in thickness. A difference below 28%, 26%, and 18% between bilateral PPL, DPL, and AT, respectively, falls within the 95^th^ percentile.

## Background

Enthesitis, inflammation of areas of attachment of ligaments, tendons, capsule, and fascia to bone, is a characteristic finding of Enthesitis Related Arthritis (ERA), subgroup of Juvenile Idiopathic Arthritis (JIA) characterized by enthesitis and arthritis. Enthesitis is typically diagnosed on physical examination by eliciting tenderness to palpation of entheseal sites. However, this finding may not be specific for inflammation as studies have suggested that entheseal tenderness may be confounded by fibromyalgia tender points [1-3]. Computed tomography and plain radiographs can only detect the bony changes of enthesitis and cannot evaluate soft tissue. MRI is impractical, requiring a specialized facility out of clinic. It is costly, time-consuming, and may require sedation. Recent studies suggest that ultrasound may be useful for the detection of enthesitis and monitoring treatment of enthesitis in adults [4-6]. Using ultrasound to detect enthesitis may be more objective and reliable than physical examination. Compared with other imaging modalities, ultrasound is cost-effective and quick. It does not require sedation and allows for dynamic and static evaluation. The OMERACT (Outcome Measure in Rheumatology in Clinical Trials) ultrasound group published consensus definitions of ultrasound pathology, which included enthesopathy. Enthesopathy is defined as an “abnormally hypoechoic (loss of normal fibrillar architecture) and/or thickened tendon or ligament at its bony attachment (may occasionally contain hyperechoic foci consistent with calcification), seen in 2 perpendicular planes that may exhibit Doppler signal and/or bony changes including enthesophytes, erosions, or irregularity [7]”.

Applying the OMERACT sonographic definition of enthesopathy to children poses several issues. Literature on the musculoskeletal ultrasound findings in the healthy pediatric population is lacking. Normal entheseal thickness has not been characterized in children. While the ultrasound technique of using the unaffected contralateral side as a control for comparison in children has been described [8], variability of entheseal thickness between contralateral sides has not been determined. This was a pilot study to explore the sonographic findings of lower extremity entheses in healthy children.

## Methods

### Patients

Consecutive patients or patients’ siblings, ages 5 to 18 years, referred to the Pediatric Orthopedic Surgery or Adolescent Medicine outpatient clinics were recruited if they were healthy and denied any lower extremity or back pain. Exclusion criteria included a history of osteochondritis, arthritis, connective tissue disease, psoriasis, inflammatory bowel disease, or any spinal or lower extremity abnormalities. No patients excluded because they were screened by the orthopedic surgeon or adolescent medicine physician to meet inclusion and exclusion criteria. Less than 5% declined participation due to time constraints or not wanting to participate. Demographic data collected included age, gender, race, weight, height, and Body Mass Index (BMI).

### Ultrasound examination

Ultrasound examinations were performed using an Esaote MyLab Class C (Esaote CA, USA) with a 6-18 MHz linear array probe by one pediatric rheumatologist trained in musculoskeletal ultrasound (CL). Grey-scale images were obtained according to a standardized protocol based on EULAR (European League Against Rheumatism) guidelines [9].

**Figure 1:**
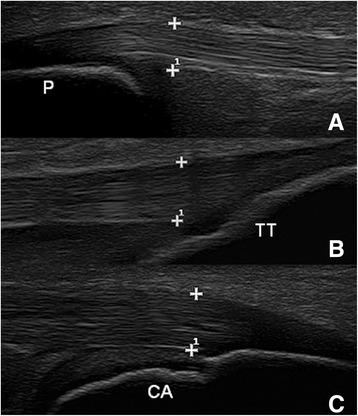
Entheseal thickness measurements. Measurements taken from a 10 year old female at the PPL **(A)**, DPL **(B)**, and AT **(C)** at the site of insertion onto bone/cartilage, perpendicular to the tendon/ligament fibers (P = patella, TT = tibial tuberosity, CA = calcaneus).

Three entheseal sites were evaluated bilaterally: proximal patellar ligament attachment to the patella (PPL), distal patellar ligament attachment to the tibial tuberosity (DPL), and Achilles tendon insertion into the calcaneus (AT). Each enthesis was examined with grey scale imaging in transverse and longitudinal planes and was interpreted by the sonographer. The PPL and DPL were evaluated with the patient lying supine on the examination table with the knee in neutral position approximated at 30° flexion by placing a rolled towel under the knee. Although a goniometer was not used to measure the degree of flexion, an appropriately sized towel was folded to varying heights to approximate 30° flexion. The AT was evaluated with the patient lying prone on the examination table with the feet beyond the edge of the table with the ankle in neutral position at 90° dorsiflexion. Entheseal thickness for each site was measured perpendicular to the tendon/ligament fibers by electronic calipers on longitudinal plane at the site of insertion onto the bone/cartilage (Figure 1). Transverse views were used to evaluate entheseal quality. It is more technically difficult to measure thickness on transverse plane which would lead to less accuracy. The following characteristics of the enthesis were assessed: hypoechogenicity (loss of fibrillar pattern), enthesophytes, erosions, and calcifications. A single sonographer was used to reduce variability in a pilot descriptive project.

### Analysis

Descriptive techniques were used to summarize the ultrasound measurements and entheseal quality. Correlations were examined with Pearson correlation coefficient, univariate analysis with t-test, and multivariate models with general estimating equation techniques (GEE). All statistical tests were performed with STATA 12.0 software for Windows (StataCorp LP, College Station, TX, USA).

## Results

### Demographics

702 entheses were examined in 117 subjects between February 2012 and May 2013. Table 1 shows the demographics of the study population; the majority of patients were male and White. Mean age was 10 years. Weight, height and BMI were recorded when available. Weight ranged from 16.8-120.3 kg (n = 104), height ranged from 106.7-194 cm (n = 87), and BMI ranged from 14-40.9 kg/m^2^ (n = 87).

**Table 1:** **Demographics**

**Subjects**	**N (%)**
Age (years)	117 (100)
5-6	22 (19)
7-8	27 (23)
9-10	22 (19)
11-12	12 (10)
13-14	20 (17)
15-16	7 (6)
17-18	7 (6)
BMI	87 (100)
<18 kg/m^2^	36 (41)
18-24 kg/m^2^	36 (41)
>24 kg/m^2^	15 (18)

### Entheseal thickness by age

**Figure 2:**
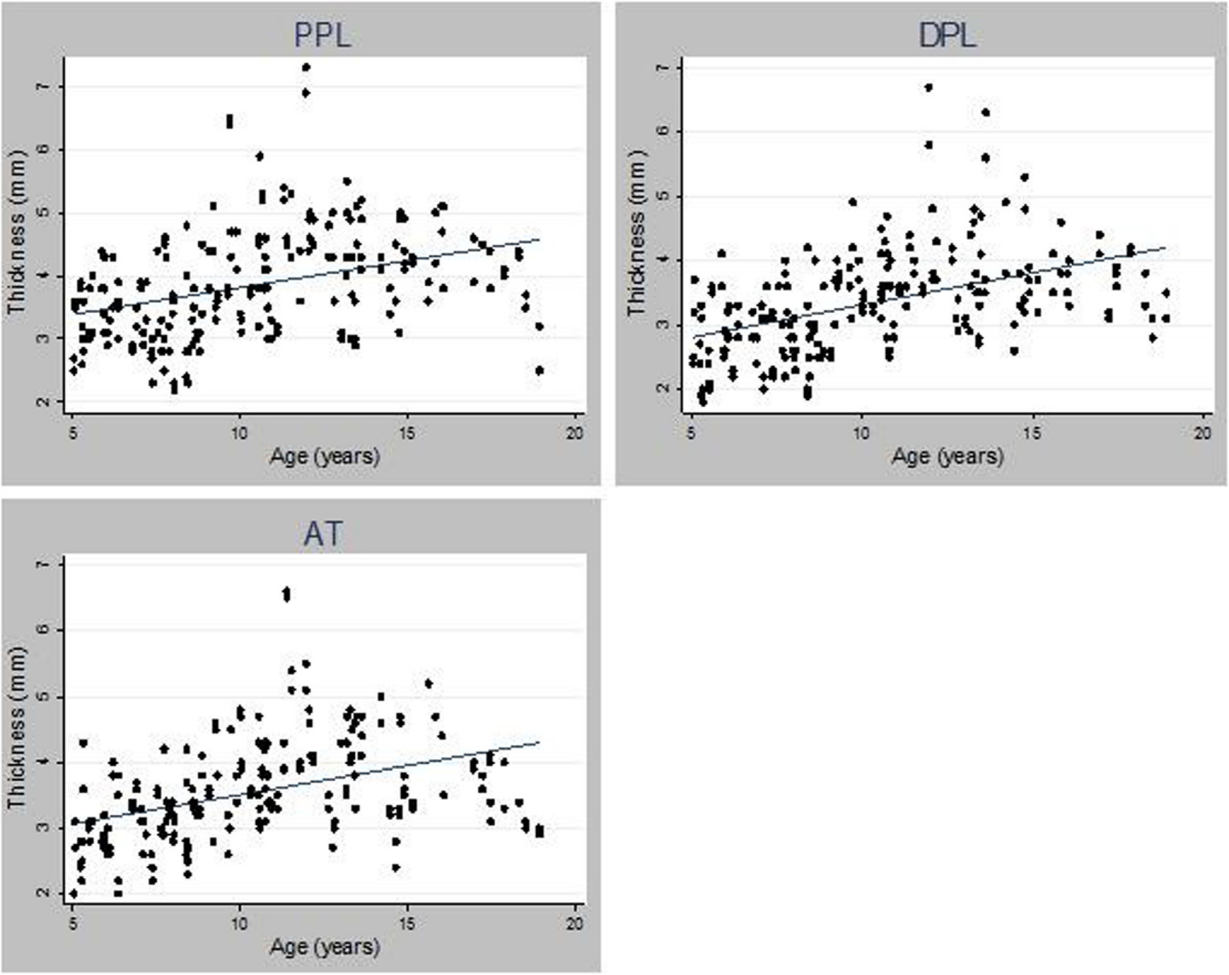
Relationship between entheseal thickness and age.

Entheseal thickness of PPL, DPL, and AT ranged from 1.8 to 7.3 mm depending on anatomic site and age group. There was large variability both within anatomic site and age group (Table 2). Comparison of age and entheseal thickness demonstrated only moderate positive correlation (r = 0.36, 0.47, and 0.41 for the PPL, DPL, and AT, respectively (Figure 2), p < 0.0001). Correlation between thickness and age did not change significantly after stratifying by gender. Multivariate models constructed to control for gender, race, and entheseal site revealed that entheseal thickness at all 3 sites increased by 0.09 mm for every additional year of age (n = 117, 103 95% CI = 0.06-0.12 mm, p = <0.0001). When adjusted for weight, the increase in thickness by age was no longer significant.

**Table 2:** **Entheseal thickness by age**

**Age (years)**	**N**	**PPL**		**DPL**		**AT**	
		**Mean ± SD (range) (mm)**	**25,50,75** ^**th**^ **%ile**	**Mean ± SD (range) (mm)**	**25,50,75** ^**th**^ **%ile**	**Mean ± SD (range) (mm)**	**25,50,75** ^**th**^ **%ile**
5-6	22	3.4 ± 0.5 (2.5-4.4)	3.1, 3.5, 3.8	2.8 ± 0.6 (1.8-4.1)	2.4, 2.8, 3.3	3 ± 0.5 (2-4.3)	2.7, 2.9, 3.3
7-8	27	3.2 ± 0.7 (2.2-4.8)	2.8, 3.1, 3.6	2.9 ± 0.5 (1.9-4.2)	2.5, 2.8, 3.1	3.1 ± 0.5 (2.2-4.2)	2.8, 3.2, 3.4
9-10	22	4.1 ± 0.8 (3-6.5)	3.5, 3.8, 4.6	3.6 ± 0.6 (2.5-4.9)	3.2, 3.6, 4	3.7 ± 0.6 (2.6-4.8)	3.4, 3.8, 4.1
11-12	12	4.7 ± 1 (3.1-7.3)	4.3, 4.6, 5	4 ± 0.8 (2.9-6.7)	3.5, 3.8, 4.2	4.2 ± 1.1 (2.7-6.6)	3.4, 4.1, 5
13-14	20	4 ± 0.7 (2.5-5.5)	3.3, 4.1, 4.6	3.8 ± 0.8 (2.7-6.3)	3.3, 3.7, 4.4	4 ± 0.7 (2.4-5)	3.4, 4.1, 4.6
15-16	7	4.3 ± 0.5 (3.6-5.1)	3.9, 4.2, 4.7	3.7 ± 0.5 (2.6-4.6)	3.4, 3.8, 4.1	4 ± 0.7 (3-5.2)	3.4, 4, 4.7
17-18	7	3.9 ± 0.6 (2.5-4.5)	3.7, 4, 4.4	3.5 ± 0.4 (2.8-4.2)	3.1, 3.6, 3.9	3.4 ± 0.4 (2.9-4.1)	3.1, 3.4, 3.8

This table shows entheseal thickness stratified by two-year age groups. The mean with standard deviations and interquartile ranges are reported. The minimum and maximum thickness (min-max) in each age group is also reported.

PPL = proximal patellar ligament insertion thickness, DPL = distal patellar ligament insertion thickness AT = Achilles tendon insertion thickness.

### Entheseal thickness by weight, height, and BMI

**Figure 3:**
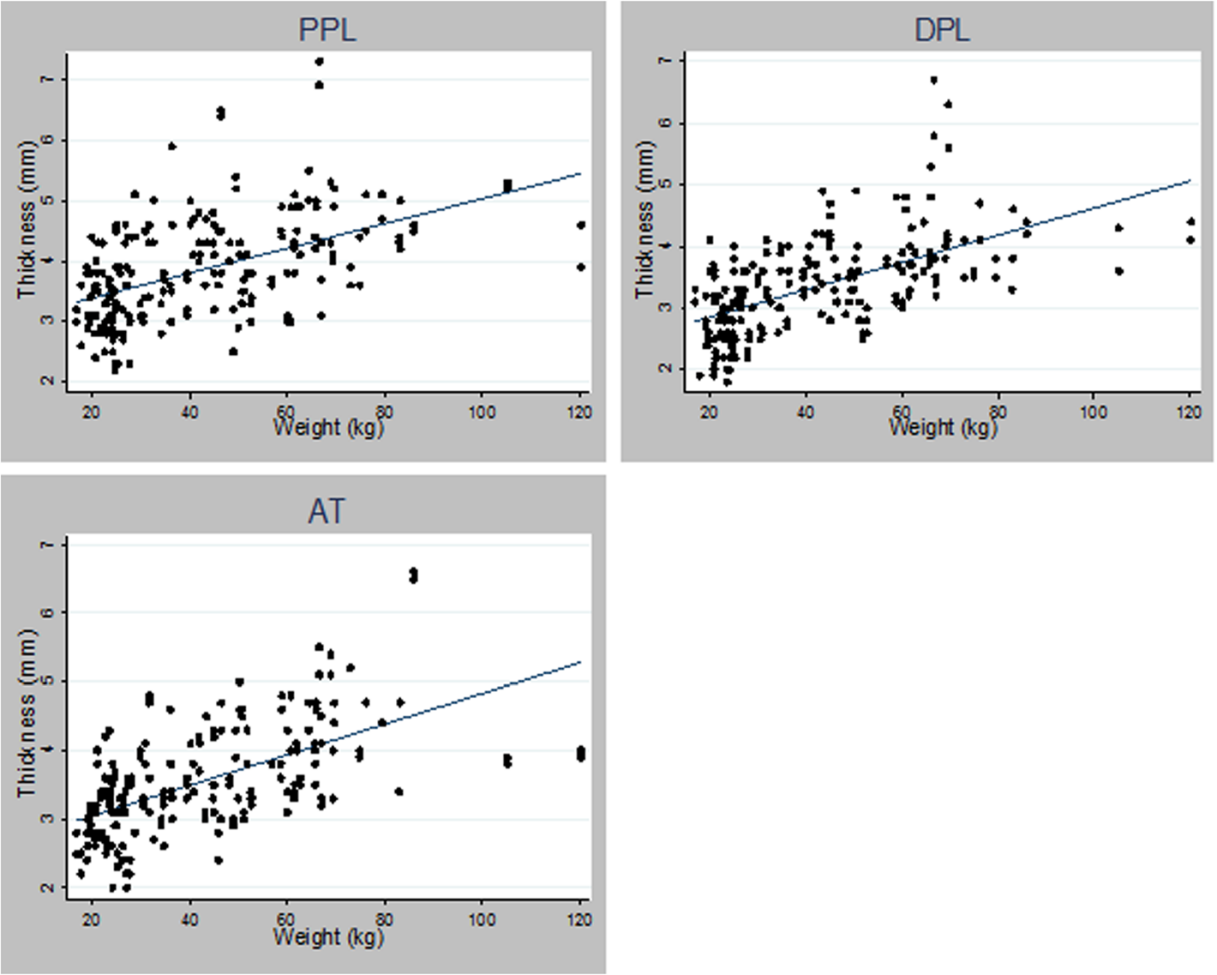
Relationship between entheseal thickness and weight.

Entheseal thickness demonstrated moderate correlations with weight, height, and BMI. The best correlation was seen with weight, r = 0.50, 0.59, and 0.60 for the PPL, DPL, and AT, respectively, p < 0.0001 (Figure 3). Table 3 demonstrates entheseal thickness stratified by weight. Entheseal thickness at all 3 sites increased by 0.02 mm for every additional kilogram of weight (n = 104, 95% CI = 0.02-0.03 mm, p = <0.0001) and by 0.07 mm for every additional 1 kg/m^2^ in BMI (n = 87, 95% CI = 0.04-0.09, p < 0.0001) when adjusted for age, gender, race, and entheseal site.

**Table 3:** **Entheseal thickness by weight**

**Weight**	**n**	**PPL (mm)**		**DPL (mm)**		**AT (mm)**	
**(kg)**		**Mean± SD (range)**	**25,50,75%ile**	**Mean± SD (range)**	**25,50,75%ile**	**Mean± SD (range)**	**25,50,75%ile**
>25	28	3.2±0.5 (2.2-4.4)	2.9, 3.2, 3.6	2.7±0.5 (1.8-4.1)	2.4, 2.6, 3.1	3.1±0.5 (2-4.3)	2.7, 3, 3.4
25-34.9	20	3.7±0.7 (2.3-5.1)	3.1, 3.7, 4.4	3.2±0.5 (2.2-4.1)	2.8, 3.2, 3.6	3.2±0.6 (2-4.8)	2.7, 3.2, 3.5
35-44.9	13	4.1±0.7 (3-5.9)	3.6, 4.1, 4.6	3.7±0.5 (2.7-4.9)	3.3, 3.6, 4	3.7±0.5 (3-4.6)	3.4, 3.8, 4.1
45-54.9	15	4.1±1 (2.5.1-6.5)	3.3, 3.8, 4.5	3.4±0.6 (2.5-4.9)	3, 3.3, 3.6	3.6±0.7 (2.4-5)	3.1, 3.5, 4.3
55-64.9	11	4.3±0.7 (3-5.5)	3.8, 4.4, 4.9	3.8±0.5 (3-4.8)	3.3, 3.7, 4	3.9±0.5 (3.1-4.8)	3.5, 3.8, 4.3
65-74.9	11	4.5±1 (3.1-7.3)	3.9, 4.4, 4.9	4.3±1 (3.2-6.7)	3.6, 3.9, 4.8	4.4±0.7 (3.2-5.5)	3.9, 4.3, 5.1
>75	6	4.6±0.4 (3.9-5.1)	4.4, 4.6, 4.9	4.1±0.4 (3.3-4.7)	3.8, 4.1, 4.4	4.6±1 (3.4-6.6)	4, 4.6, 4.7

This table shows entheseal thickness stratified by 10 kilogram intervals. The mean with standard deviations and interquartile ranges are reported. The minimum and maximum thickness (range) in each weight group is also reported.

PPL= proximal patellar ligament insertion thickness, DPL=distal patellar ligament insertion thickness AT=Achilles tendon insertion thickness.

### Gender and ethnic effects on entheseal thickness

Entheseal thickness overall was significantly larger in males than females even after adjusting for entheseal site, age, race, weight, and BMI (p = 0.01). Multivariate analysis demonstrated that entheseal thickness of Hispanics was significantly smaller compared with non-Hispanic Whites (p = 0.03). However, the larger thickness of the entheses observed in African Americans lost its significance when adjusted using the same model (p = 0.25).

### Entheseal thickness variability in contralateral sites

The difference in thickness between bilateral sites for the PPL ranged from 0 -1.9 mm (mean ± SD (standard deviation) =0.40 ± 0.35mm), from 0-1.9mm (mean ± SD = 0.34 ± 0.31mm) for the DPL and from 0-0.8 mm (mean ± SD = 0.20 ± 0.18 mm) for the AT. Using pair-wise correlation, Pearson’s correlation coefficient (r) for contralateral entheseal thickness was 0.80 for PPL, 0.83 for DPL, and 0.94 for AT (p < 0.0001). Likewise, a two-tailed paired t-test demonstrated no significant difference in entheseal thickness between contralateral sites of the PPL (p = 0.44), DPL (p = 0.52), and AT (p = 0.66).

**Figure 4:**
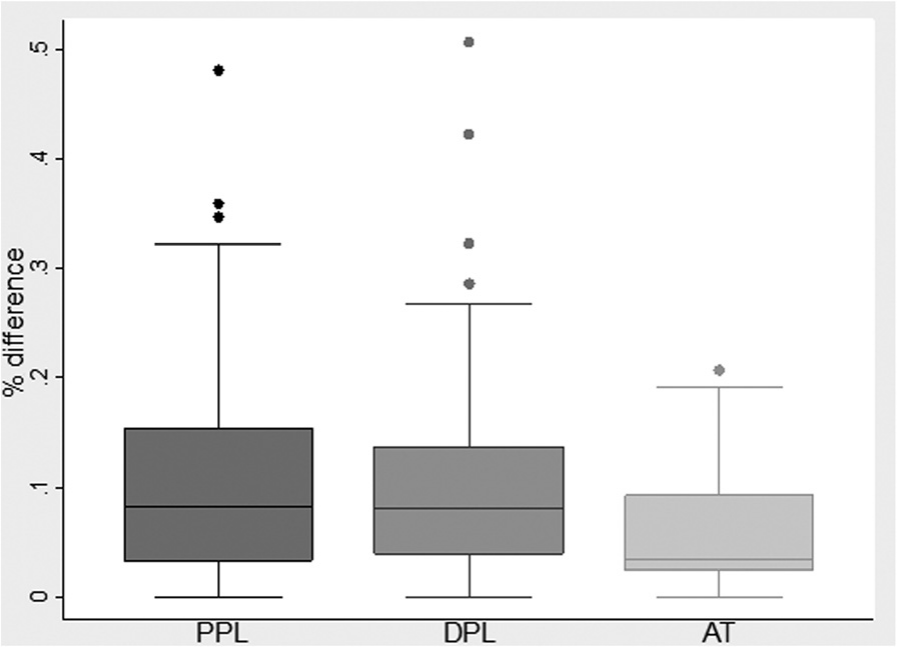
Difference in bilateral thickness by entheses.

Considering the absolute difference between sites may be larger in thicker entheses, the percent difference between contralateral sites was calculated. The mean percent differences ± SD between bilateral PPLs was 10.64 ± 9.07%, DPLs 9.86 ± 8.28%, and between ATs 5.76 ± 5.21% (range: 0-48.1%, 0-50.67%, and 0-20.69%, respectively). The distribution of percent differences was skewed towards a smaller percent difference as seen in the box and whiskers plot in Figure 4. Percent differences between bilateral sites that fell within the 95^th^ percentile of the study population were 28% for the PPL, 26% for the DPL, and 18% of the AT. Univariate and multivariate analysis demonstrated a consistent relationship between thicknesses at different entheses, with DPL and AT consistently smaller than the PPL (p < 0.0001).

### Entheseal quality

**Figure 5:**
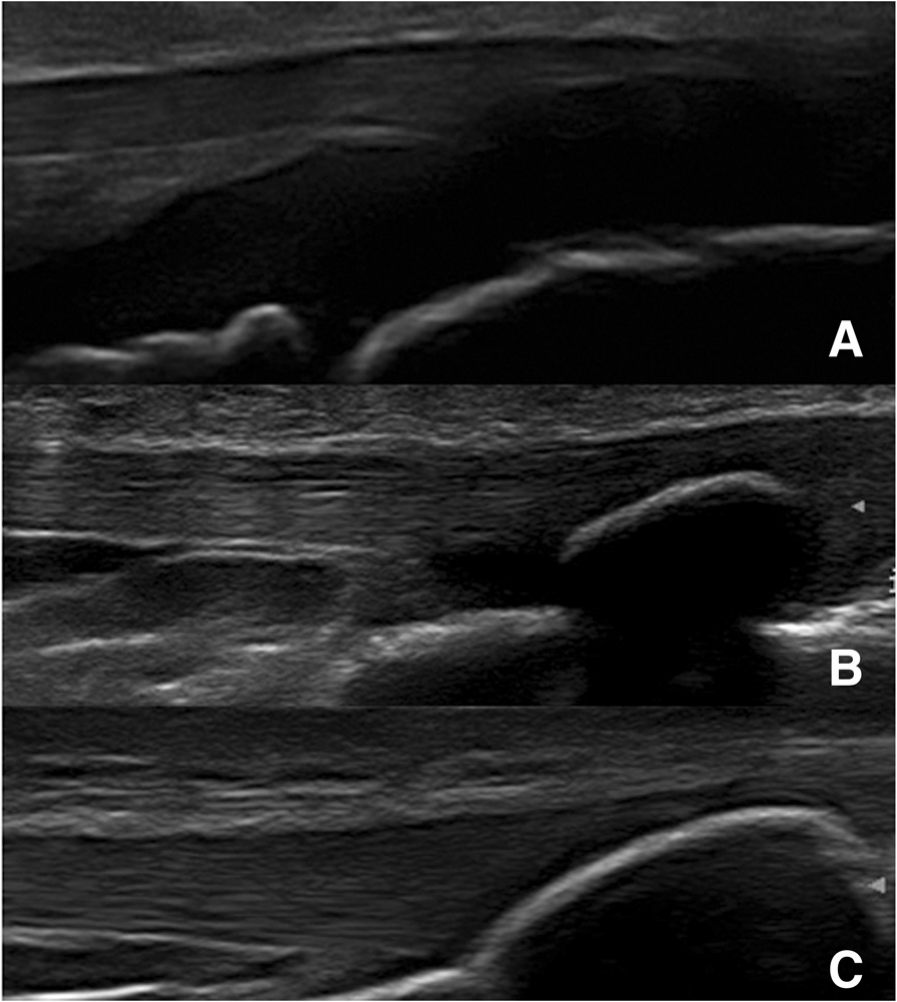
Progression of entheseal insertion. DPLs of a 6 year old **(A)**, 10 year old **(B)** and 18 year old **(C)**: progression of the entheses from completely cartilaginous to completely osseous.

**Figure 6:**
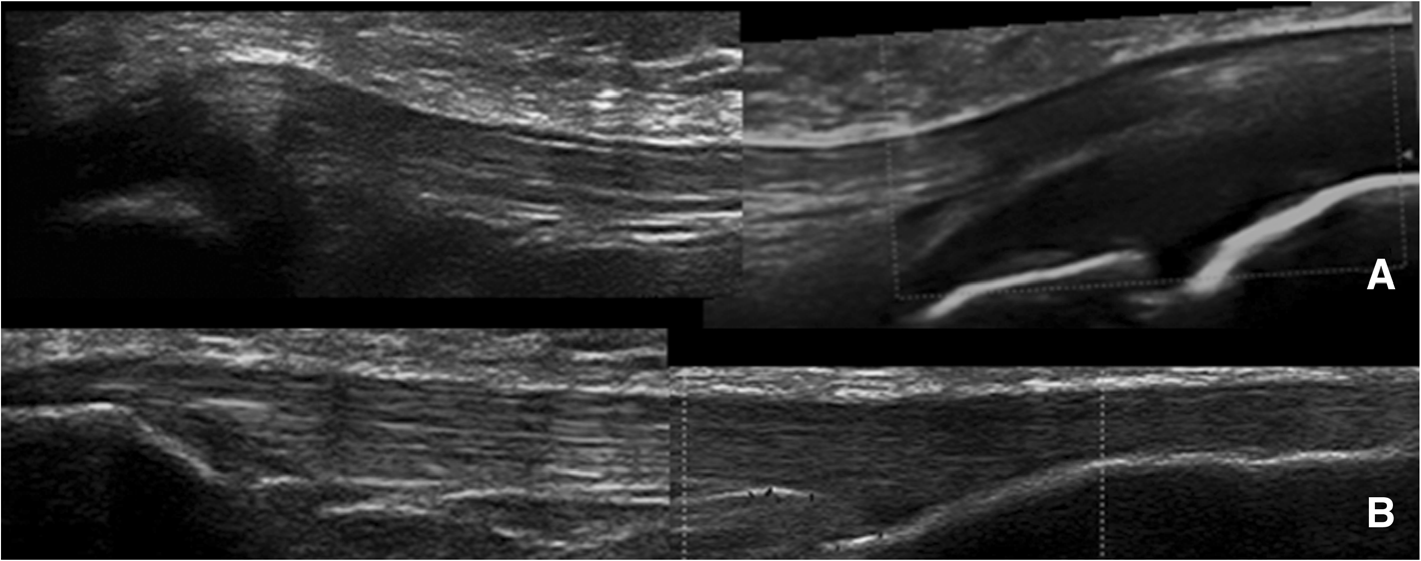
Change in patellar ligament contour with age. Patellar ligament with its proximal and distal attachments in a 6 year old male (Image **A**) and an 18 year old female (Image **B**). Note that the curved contour with concave middle and convex attachments **(A)** transitions to a linear contour with increasing age **(B)**.

Entheses in all subjects demonstrated a homogeneous fibrillar pattern as seen in Figure 1. At the interface with bone, entheses were completely cartilaginous in younger children (Figure 5A). With increasing age, the entheseal connexion transitioned to an osseous attachment (Figure 5B). The insertion sites ossified with increasing age (Figure 5C), which supports previous studies [10-12]. The transition from completely cartilaginous to completely osseous was at an earlier age in the PPL than DPL. Calcifications within the tendon/ligament or enthesophytes, which are characteristic ultrasound findings of enthesitis in adults [7], were not seen in any of the subjects. The contour of the patellar ligament varied with age. In the younger subjects, the patellar ligament had a curved route with a convex appearance at its insertion sites and a concave contour between the 2 insertion sites. With increasing age, the patellar ligament became more linear and parallel to the skin surface throughout its course (Figure 6). There were no obvious changes in the Achilles tendon contour with increasing age.

## Discussion

### Entheseal thickness

Our study is the largest to date describing the sonographic grey-scale appearance of 3 lower extremity entheses in 117 healthy children of multiple racial groups. We were able to document several important correlations between entheseal thickness and demographics as well as anthropomorphic characteristics. Entheseal thickness increased with age; however the correlation was moderate with significant variability. This supports findings of a smaller previous study by Jousse-Jouline et al. which demonstrated a positive correlation with age with a large degree of variability [13]. In our study, multivariate analysis demonstrated entheseal thickness increased by 0.09 mm for every additional year; however this significance was lost when adjusted for weight. Weight had the strongest correlation to entheseal thickness. Taken together, these data suggest that increased entheseal thickness associated with age is due to increase in weight. Boys had thicker entheses than girls. Hispanic ethnicity correlated independently and inversely with entheseal thickness.

Some degree of thickness variability between contralateral sites appears to be normal with the least amount of variability in the AT and the greatest in the PPL. The percent difference between bilateral sites at the 95^th^percentile in our healthy study population was 28%, 26%, and 18% at the PPL, DPL, and AT, respectively. Further studies are needed to confirm this observation and determine if a difference greater 30% may be used as a cutoff to define disease when comparing a symptomatic enthesis with a contralateral asymptomatic control.

### Entheseal quality

**Figure 7:**
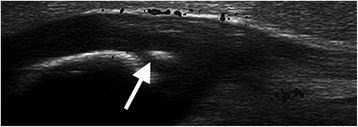
Ossification center. Longitudinal scan of the PPL showing an ossification center in the cartilaginous portion of the patella in a 5 year old boy. This should not be mistaken for an enthesophyte or calcification within the tendon/ligament.

A homogeneous, fibrillar pattern was seen in all ligaments and tendons. As in adults [7], features of entheseal quality may be important in defining enthesopathy in children. While ossification centers could be seen as hyperechoic signals within cartilage in younger children who still had a large amount of cartilage (Figure 7), these must not be mistaken for morbid processes. Doppler activity was recorded in this study and seen in some entheses of subjects; however this was not the main purpose of this study and is an area of future research.

The patellar ligament contour evolved with age from a curved appearance on longitudinal scan to a more linear contour. This supports the findings reported by Blankstein et al. that the patellar ligament changes from a “sagging rope” to a more linear structure with increasing age [12].

There are a few principal limitations to our study. Weight was unavailable in 12% and height/BMI in 26% due to incomplete data collection during clinic visits. Unfortunately, it was not part of their standard clinic visit to have a height and weight measured at each visit, and we did not have the resources to measure their height and weight at the time of the sonographic exam. Since we felt it was important to include as many subjects as possible to make conclusions in a pilot study, we included subjects without these measures. Pubertal staging was not collected, which may affect entheseal quality; however, this was not part of their standard clinic visit. Leg dominance was not recorded which may affect comparisons between bilateral entheses; however hand dominance does not always correlate with leg dominance. Leg dominance is more technically difficult to determine. The number of non-Caucasian subjects was small. Only 1 sonographer (CL) performed, measured, and interpreted all ultrasound exams. One sonographer was used to decrease variability. Because ultrasound is an operator dependent examination with possible inter-observer variability, a second sonographer would help determine reproducibility of findings. Determining inter and intra reader variability would be a goal for a future study.

## Conclusions

Our study is pilot study to explore normative sonographic data for the entheses of healthy children. Obtaining normative data will be fundamental to the sonographic assessment of disease. Further studies including Doppler findings in healthy children and comparing children with osteochondritis and enthesitis will characterize the specific sonographic features of each condition.

### Consent

Written informed consent was obtained from the patient’s guardian/parent/next of kin for the publication of this report and any accompanying images.

## Abbreviations

PPL: Proximal patellar ligament insertion
DPL: Distal patellar ligament insertion
AT: Achilles tendon insertion
ERA: Enthesitis related arthritis
JIA: Juvenile idiopathic arthritis
OMERACT: Outcome Measure in Rheumatology in Clinical Trials
BMI: Body mass index
EULAR: EUropean League Against Rheumatism
P: Patella
TT: Tibial tuberosity
CA: Calcaneus
GEE: General estimating equation techniques
SD: Standard deviation
